# Clinical and Research Insights from Pre-Emptive Early Intervention for Neurodevelopmental Disorders: A Case Series

**DOI:** 10.3390/children12111489

**Published:** 2025-11-04

**Authors:** Giulia Purpura, Silvia Annunziata, Stefania Biancardi, Michelle Brivio, Camilla Caporali, Giulia Mantegazza, Elena Piazza, Alice Restelli, Anna Cavallini

**Affiliations:** 1 IRCCS Fondazione Don Carlo Gnocchi, Via Capecelatro 66, 20148 Milano, Italy; gipurpura@dongnocchi.it (G.P.); sbiancardi@dongnocchi.it (S.B.); mibrivio@dongnocchi.it (M.B.); ccaporalli@dongnocchi.it (C.C.); gmantegazza@dongnocchi.it (G.M.); epiazza@dongnocchi.it (E.P.); arestelli@dongnocchi.it (A.R.); ancavallini@dongnocchi.it (A.C.); 2 School of Medicine and Surgery, University of Milano-Bicocca, 20900 Monza, Italy

**Keywords:** early intervention, neurodevelopmental disorders, autism spectrum disorder, family-centered care, case report

## Abstract

**Highlights:**

**What are the main findings?**

- Early and pre-emptive structured interventions may have the potential to enhance long-term neurodevelopmental outcomes.
- Siblings of children with ASD may develop atypical trajectories in several developmental domains.

**What is the implication of the main finding?**

- Considering all developmental domains is a key point of early interventions for children at risk of neurodevelopmental disorders.
- A multidimensional approach with the active involvement of parents is crucial for the effectiveness of early intervention programs.

**Abstract:**

**Background:** Siblings of children with autism spectrum disorder (ASD) are considered biologically at risk of developing neurodevelopmental disorders (NDDs) that may involve sensorimotor, adaptive, and social–communication domains. Previous studies have highlighted the potential value of early intervention programs during the first year of life, when symptoms may not yet be evident. This study explores the impact of pre-emptive and early interventions on the developmental trajectories of infants at risk for NDDs. **Methods:** This case-series study included four children (one at low risk and three at high risk) who participated in the ERI-SIBS Project (Early Recognition and Intervention in Siblings at High Risk for Neurodevelopmental Disorders), an ongoing, innovative, and ecological early recognition and intervention program for siblings of children with ASD. Intervention frequency was personalized based on the presence or absence of early risk indicators and continued for six months. Data on global functioning, social-communication abilities, and mother–child interactions were collected over one year. **Results:** Qualitative analysis revealed four distinct developmental trajectories and treatment responses, emphasizing the need for a multidimensional approach and the active engagement of parents in the intervention process. **Conclusions:** Findings from this case series provide an in-depth understanding of how biological and environmental factors may interact to influence the outcomes of early interventions in children at risk for NDDs. These results underscore the importance of early, individualized, and family-centered approaches to support optimal developmental outcomes.

## 1. Introduction

Early interventions (EIs) refer to the targeted and timely provision of systematic therapeutic strategies from birth to 36 months. EIs aim to prevent or minimize sensory–motor, cognitive, social, and emotional impairments in infants and young children disadvantaged by biological or environmental risk factors [1,2]. The growing interest in clinical practice and research for these types of interventions has been strongly related to the enhanced knowledge about the neuroplasticity of the young brain, which provides the opportunity for the most significant impact of EI on long-term outcomes of children at high risk of neurodevelopmental disorders (NDDs) [3]. Regarding the underlying mechanisms of EI, animal and human models suggest that providing environmental enrichment at an early stage of age can significantly impact the developmental trajectories of these populations due to triggering a marked acceleration in the maturation of neural circuits and their correlated functions [4,5]. Multisensory and social enrichment in this period acts on experience-dependent structural synaptic plasticity that is abundant in the developing brain and is thought to represent the neurobiological substrate for learning and memory formation [6]. Although some issues about the administration of EI need to be better investigated, participating in early intervention programs gives children with or at risk of developmental disabilities and their families a chance to have an individualized program that can identify particular and unique needs and reduce the long-lasting impact of these conditions.

Recently, this line of research has expanded to investigate whether this early approach can also impact social skills, thereby preventing impairment in interaction and communication domains. Interesting results in this aspect came from animal models, in which profound and long-term beneficial effects of early social environmental stimulation were found, encouraging nonpharmacological interventions to improve social defects in neurodevelopmental diseases [7,8]. Regarding clinical implications of this research line, Nelson et al. [9] highlighted that this aspect must also be taken into consideration in the case of autism spectrum disorders (ASD), in which deviations from typical neurodevelopmental trajectories may appear early in childhood and impact social and interaction skills and sensory–motor behaviors. EI may play a crucial role in ASD for three reasons: (i) functional connectivity patterns in infants, regardless of autism risk, change in the first years of life, (ii) early connectivity patterns between 3 and 6 months can predict autism symptoms in toddlers, and (iii) experiences influence connectivity, leading to increased and more distributed connections for gross motor and social communication behaviors by 24 months compared to one year earlier [9].

Based on these insights, the role of pre-emptive interventions in children considered at familial risk for neurodevelopmental disorders (NDDs) has recently begun to emerge in the scientific literature. The term “pre-emptive intervention” refers to early strategies explicitly addressing the developmental challenges of infants under 12 months of age, whether or not they display subtle signs of neurodevelopmental vulnerability. Pre-emptive intervention differs from traditional early intervention in that it targets infants at high risk for NDDs before overt symptoms emerge. While early intervention typically addresses developmental concerns once they are evident, pre-emptive approaches aim to mitigate the onset of these concerns through timely, targeted strategies. Research indicates that pre-emptive intervention can lead to improved developmental outcomes, including reduced severity of ASD symptoms and a lower likelihood of an ASD diagnosis by age 3 [10]. This need has arisen from recent research focusing on the identification of early signs of autism in the first year of life, with the aim of developing effective screening and early intervention programs for infants considered biologically at risk. For example, siblings of children with ASD are at increased risk of atypical developmental trajectories, even if they do not meet ASD diagnostic criteria [11]. In these children, reduced attentiveness to parents, diminished affective signaling, poorer coordination of communication, sensory–motor differences, and other signs of neurodevelopmental vulnerability have been observed from six months of age in those later diagnosed with ASD. Based on these observations, it has been hypothesized that such effects may be evident even in the first months of life [12]. These findings underscore the necessity of advancing beyond traditional early intervention to incorporate pre-emptive strategies that proactively address developmental vulnerabilities.

The cases presented in this study aim to examine and reflect on the impact of a pre-emptive early intervention program on the developmental trajectories of infants at risk for neurodevelopmental disorders (NDDs). The children included in this study participated in the ERI-SIBS protocol [13]. The Early Recognition and Intervention in SIBlingS at High Risk for Neurodevelopmental Disorders (ERI-SIBS) project is an ongoing, innovative, and ecologically grounded program designed specifically for siblings of children with ASD. Unlike other early intervention programs for this high-risk population, ERI-SIBS employs a multidimensional approach to enrich the child’s environmental experience across socio-communicative, sensorimotor, cognitive, linguistic, and affective domains. The main strengths of the ERI-SIBS project lie in its comprehensive approach, which does not target solely socio-communicative skills or autism-related symptoms, but considers the child’s overall developmental profile. In addition, the program gives support in promoting development even to siblings who may not yet exhibit clear signs of risk for neurodevelopmental disorders before the first year of life. Data from this case series may offer valuable insights into the importance of adopting a multidimensional and multidisciplinary approach in pre-emptive early interventions, given that siblings of children with ASD may follow diverse developmental trajectories, even in the absence of ASD-specific concerns.

The primary aim of this study is clinical, focusing on the practical applicability of this type of intervention within healthcare services. The study emphasizes that tailoring the habilitative treatment to the child’s individual characteristics, as well as to the specific needs and context of the family, is crucial for maximizing its effectiveness. While no single intervention is effective for all children at risk of neurodevelopmental disorders, certain evidence-based principles should always be considered. By highlighting these aspects, this study aims to provide readers with a framework for understanding how personalized, family-centered approaches can enhance early intervention outcomes.

## 2. Materials and Methods

### 2.1. Participants

The case series described in this study is part of a wider project (ERI-SIBS) being carried out from 2024 to 2027 in the Child Neuropsychiatry and Rehabilitation Section of the Scientific Institute Santa Maria Nascente of the Don Gnocchi Foundation in Milan, Italy. The reported investigation was conducted in accordance with the principles outlined in the Declaration of Helsinki. Informed consent was obtained from all participating children’s parents in written form. Ethical approval was obtained from Comitato Etico Territoriale 4: C.E. “B-INT” 4 (CET 14/24). The trial has been registered on ClinicalTrials.gov (identifier NCT06512649).

For recruitment, children were included if they met the following criteria: (i) having one or more biological siblings; (ii) being no older than 10 months at the first assessment; (iii) exhibiting a normal neurological examination; (iv) having parents who provided informed consent; and (v) having parents with sufficient proficiency in the Italian language. Exclusion criteria were: (i) a known or suspected diagnosis of neurological, sensory, or genetic disorders at the time of recruitment; (ii) parents not fluent in Italian; and (iii) prior enrollment in developmental disorder treatment programs.

The participants described in this study are four male infants who had completed the ERI-SIBS program and were between 7 and 8 months of age at the time of recruitment. These cases were selected explicitly from our sample because each appeared to represent a distinct developmental trajectory, while sharing comparable socio-cultural backgrounds, similar attendance at scheduled sessions (for assessments and interventions), and analogous demographic characteristics (e.g., gender, gestational age, and age at recruitment).

Among these participants, Case 1 was a low-risk infant, that is, a sibling of a child with typical development, while the other three children (Case 2, Case 3, and Case 4) were classified as at high risk because they were siblings of children with ASD. All children were born at full-term pregnancy, and delivery was uneventful. At recruitment, a clinical examination was performed on all infants. It included a neurologic examination, neuropsychological assessment, and mother–infant interaction assessment. Neurological examination revealed no major sensory or neuromotor impairment in all children. For other details, see Table 1.

According to the ERI-SIBS protocol [13], based on the results of clinical examination, recruited infants were collocated into one of these three groups:

*Group 1*—Clinical Monitoring Group (CM): Siblings of TD children with no signs of concern;
*Group 2*—Active Monitoring Group (AM): Siblings of ASD children with no signs of concern;
*Group 3*—Early Intervention Group (EI): Siblings classified as “with signs of concern” at the baseline evaluation.

Scores on the Griffiths Scales of Child Development, 3rd Edition (Griffiths-3) and the Communication and Symbolic Behavior Scales—Infant–Toddler Checklist (CSBS-ITC) were the primary tools for assigning infants to one of the three study groups (see Section 2.2). Both instruments provide scores based on normative data, allowing comparisons with standardized developmental expectations for age. Infants with a developmental quotient below 85 on any Griffiths-3 subscale and/or at least one score above the CSBS-ITC “concern” threshold (scores ≤ 15 percentile) were assigned to Group 3.

### 2.2. Outcome Measures

Assessments were conducted at three time points: at baseline, when the parents provided their consent within eight months of the child’s life (T0), six months after the start of monitoring/intervention (T1), and 12 months after the start of monitoring/intervention (T2). For this study, we reported only some outcome measures established in the ERI-SIBS project that were in line with the aim of this investigation. Outcome measures were:

- *Griffiths Scales of Child Development, 3rd Edition (Griffiths-3).* Griffiths-3 is considered the gold standard for assessing overall child development in children aged 0–6 years. It provides a comprehensive profile of a child’s strengths and needs across five developmental domains: foundations of learning, language and communication, eye–hand coordination, personal–social–emotional, and gross motor skills [14]. The Griffiths-3 yields standardized scores based on normative data, allowing each child’s performance to be compared with age-matched peers and identifying areas that may require closer monitoring or intervention. In this study, the test was administered at all three assessment time points, enabling the evaluation of developmental trajectories over time and the detection of early signs of neurodevelopmental vulnerability relative to typical development.
- *The Communication and Symbolic Behavior Scales—Infant–Toddler Checklist (CSBS-IT-C).* It is a standardized developmental screening tool that measures seven key predictors of language and communication in children aged 6–24 months [15]. The checklist can be administered to parents in an interview format, with explanations to clarify each item. Completion typically requires 5–10 min. Significantly, the CSBS-ITC scores are based on normative data, which allows each child’s performance to be compared with age-matched peers. Scores exceeding established “concern” thresholds indicate potential early signs of ASD, guiding the identification of infants who may benefit from closer monitoring or intervention. In this study, the CSBS-ITC was administered at all three assessment time points, enabling both the detection of early signs of concern and the tracking of developmental trajectories relative to standardized norms.
- *Autism Diagnostic Observation Schedule, Second Edition (ADOS-2)- Toddler Module*. The ADOS-2 is a semi-structured, standardized assessment of communication, social interaction, play/imaginative use of materials, and restricted/repetitive patterns of interest to assess the presence of ASD symptoms [16]. The Toddler Module is used specifically for 12–30-month-old children. The administration involves direct observation using hierarchical manualized procedures and progressive prompts. Every behavior/symptom is assessed using a Likert scale (0–3) and coded on an algorithm based on DSM-5 diagnostic criteria. This test was administered only at T2.
- *Parent Interactions with Children: Checklist of Observations Linked to Outcomes (PICCOLO)*. This is an observational measure in which a mother–child interaction is video recorded, and trained observers code specific parenting behaviors known to predict children’s early social, cognitive, and language development [17]. Specifically, the PICCOLO examines four domains of parenting, including behaviors such as affection, responsiveness, encouragement, and teaching. Each domain is scored on a 0–2 Likert scale. This project used the PICCOLO at all three time points.
- *Satisfaction Survey:* Parents of children in Groups 2 and 3 were asked to complete an anonymous, project-specific survey immediately following the intervention (T1) to assess their satisfaction with the services provided between T0 and T1. The questionnaire takes approximately 5 min to complete and consists of 15 items, each rated on a 5-point scale (from 1, “not at all,” to 5, “very much”).

### 2.3. Interventions

As expected from the ERI-SIBS protocol, the child (Case 1) allocated to Group 1—Clinical Monitoring—did not receive any treatment but only clinical evaluations at the defined time points. On the contrary, children in Group 2—Active Monitoring (Case 2) and Group 3—Early Intervention (Cases 3 and 4) received training for the first six months. The clinicians involved in the study have substantial experience working with families of children with early neurodevelopmental difficulties. Weekly meetings were carried out for peer clinical supervision within the context of a multidisciplinary team. All intervention sessions (Groups 2 and 3) were recorded and securely stored on a device owned by the principal investigator, enabling thorough supervision and review by the multidisciplinary team.

- **Active Monitoring Group:** This multimodal and ecological approach has been planned for siblings of children with ASD who do not show early signs of concern. Following the environmental enrichment paradigm, this pre-emptive program has been structured explicitly for HR siblings. This choice is based both on the evidence that siblings of ASD children without early signs of concern can have a higher probability of developing later other neurodevelopmental disorders (for example, language disorder) [18] and on the data that supported as also no-ASD high-risk siblings show different patterns of behaviors in temperament, motor, social and linguistic domains in comparison to low-risk children, also in the absence of a specific diagnosis [19,20,21,22,23]. For these reasons, children in this group are enrolled in 90-min sessions monthly for 6 months. Sessions are performed by an expert neuro and psychomotor therapist for developmental age (TNPEE) [24], supported by a child neuropsychiatrist or psychologist. A parent is always present and actively involved in the session. During sessions, by using play and following the self-initiated behaviors and interests of the child, professionals and parents can share and implement new strategies to promote the achievement of developmental milestones and the child’s active participation in everyday activities. Some practical advice for managing children at home can also be given to parents with more significant concerns. More details about “Active Monitoring” are reported in Annunziata et al., 2025 [13].
- **Early Intervention Group:** The early intervention program aims to promote postural–motor, socio-communicative, and cognitive skills using a multidimensional, naturalistic, and family-centered approach. The intervention has been planned by considering the child’s sensory profile and individualizing and tailoring objectives and strategies to each child’s functioning and needs. This approach has been ideated and implemented based on scientific literature that suggests that ASD siblings have a higher risk of developing ASD or other neurodevelopmental disorders [18,25], but also on data that indicate early sensory–motor development may have important prognostic implications in ASD [26,27,28,29]. Children in this group are enrolled in a 90-min parent-coaching session once a week for six months, led by an expert neuro and psychomotor therapist for developmental age (TNPEE) [24] and accompanied by a child neuropsychiatrist or psychologist. A parent (preferably the mother) is always present and actively involved in the intervention. During the sessions, clinicians work with parents to identify ways to incorporate the same strategies into daily life. Also, they encourage parents to organize playful sessions at home for regular stimulation, with a goal dose of 20–30 min per day, according to their child’s availability. Sessions may also contain a small psychoeducation component on common early parenting challenges such as sleep, crying, and feeding. More details about Early Intervention are reported in Annunziata et al., 2025 [13].

## 3. Results

### 3.1. Scores at Griffiths-3

The results of the four children among the three time points are reported in Table 2. At T0, the low-risk child (Case 1) and one of the high-risk children (Case 2) showed scores in the normal range (>85) in all five domains of Griffiths-3 and also in the General Developmental Quotient. Between T0 and T1, only the HR child (Case 2) performed Active Monitoring, while from T1 onward, both children started the frequency at daycare. As reported in Table 2, during the following 12 months, both children maintained a good developmental trend, and scores remained within the normal range at T1 and T2. The developmental maturation of the two children may be considered similar.

A different developmental trend was observed in Case 3. At T0, this child obtained scores <85 (below the normal range) in the GDS and all subscales of Griffiths-3. For this reason, he received Early Intervention between T0 and T1, but no improvements were observed. At T1, the physician suggested that the parents register the child for daycare and continue neuro- and psychomotor standard care within the National Health System. Parents encountered difficulties in adhering to the recommendations owing to the presence of logistical and familial constraints. As shown in Table 2, we observed a lack of progression in developmental maturation. Over time, this led to a deflection of the developmental trajectory.

Finally, in Case 4, a global improvement was observed between T0 and T1 following the Early Intervention. This HR child responded well to the intervention program in all domains of development at Griffiths-3. Indeed, while at T0, the GDS and three subscales quotients (Language and Communication, Personal–Social–Emotional, and Gross-Motor) were <85, after six months (at T1), all scores were in the normal range and >100. As in the previous case, the doctors suggested that the family enroll the child in a daycare and follow the standard neuro-psychomotor care within the National Health System to promote further development. For this reason, the child immediately started neuro-psychomotor therapy twice a week after assessment at T1. In this case, despite a slight decrease in scores of some domains of Griffiths-3, all quotients remained at T2 in the normal range, except for Gross-Motor skills.

### 3.2. Scores at CSBS-IT-C

**Figure 1:**
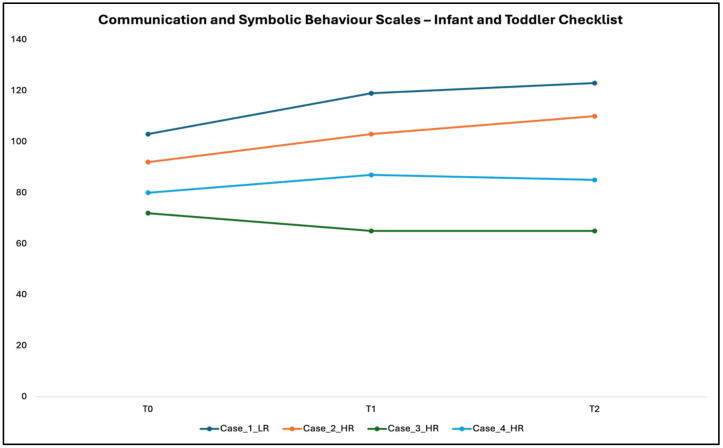
Developmental trajectories of the four children at the Global Quotient of the Communication and Symbolic Behavior Scales—Infant–Toddler Checklist.

Total scores at CSBS-IT-C showed different trajectories of maturation of social-communication abilities for each of the four analyzed children (see Figure 1).

The low-risk child (Case 1) had the highest total score at all three time points (T0: 103; T1: 119; T2: 123). Thus, this tool did not reveal any signs of concern for this child. Similarly, all scores were in the normal range for Case 2, although this child’s CSBS-IT-C quotients were much lower than those of Case 1 (more than 10 points) at all three time points (T0: 92; T1: 103; T2: 110).

Regarding Case 3, at T0, this HR child showed a general quotient under the range of typical development (70), and signs of concern were evident in the Social Composite and Speech Composite Scores. Similar to the developmental trajectories at Griffiths-3, a global worsening of scores was evident in CSBS-IT-C at T1 and T2 in comparison to T0 (T1: 65; T2: 65), with signs of concern in Social Composite, Speech Composite, and Symbolic Composite Scores at both time points.

Finally, in Case 4, the total quotient was in the limit range at T0 (80), with signs of concern only in the Speech Composite Score. After early intervention, a slight improvement was observed at T1 (87) and T2 (85). Signs of concern at T2 remained only for the Speech Composite Score.

### 3.3. Scores at ADOS-2 Toddler Module

**Figure 2:**
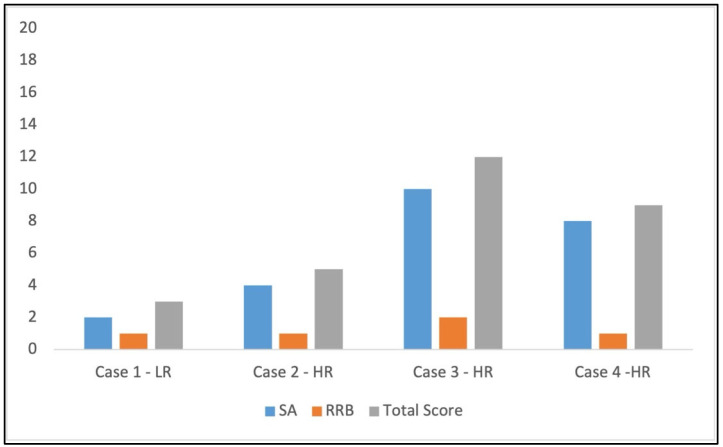
ADOS-2 scores at T2 of the four children (SA: Social Affect Score; RRB: Repetitive and Restricted Behaviors Score).

The Toddler Module of ADOS-2 was administered to the four participants at T2 (see Figure 2). In Case 1, Case 2, and Case 4, total scores were within the range for “little or no concern scores” for ASD diagnosis, although Case 4 obtained a score of 9, which is the limit of the typical range score for non-verbal children or those who use sporadic single words (0–9). Regarding Case 3, the total score (12) shows behaviors in the mild-to-moderate concern range for non-verbal children (10–13).

### 3.4. Scores at PICCOLO

Qualitative analysis was carried out for the four areas PICCOLO investigated (affection, responsiveness, encouragement, and teaching) at the three time points for every dyad (see Table 3). To ensure high coding reliability, all videos were independently scored by two evaluators who had completed specialized advanced training. Discrepancies between their ratings were resolved through discussion and by jointly reviewing the videos, ensuring consensus on all scores.

Regarding affection, which involves warmth, physical closeness, and positive expressions toward the child, the mothers of Cases 2 and 4 remained in the typical or upper range for all three time points. In Case 1, the mother’s affective behavior was in the typical range at T0 and T2, while a slight and temporary decrease under the typical range at T1 was detected. Conversely, in Case 3, affective behavior was below the typical range at all three evaluations.

Responsiveness, which includes parental responses to the child’s signals, emotions, words, interests, and behaviors, was in the normal range at T0 and T2 for the mother of Case 1, with a decrease in the borderline range only at T1. A progressive increase from values under the typical range to within the normal range was observed from T0 to T2 in mothers of Cases 2 and 4, whereas scores below the typical range were observed at all three time points in the mother of Case 3.

Regarding behaviors of encouragement that include active support to the child for exploration, initiative, curiosity, creativity, and play in autonomy, an increase in scores was evident in mothers of Cases 1, 2, and 4. In particular, the mother of Case 1 obtained scores in the typical range at T0 and T1, while the score was above the typical range at T2. The mother of Case 2 showed a score compatible with the behavior of encouragement below the typical range at T0, in the typical range at T1, and above the typical range at T2. Similarly, the mother of Case 4 shows a progressive improvement from T0, when the score was below the typical range, to T2, when it reached the typical range. Also, for the encouragement behaviors, the scores of the mother of Case 3 were below the typical range at all time points.

Finally, regarding teaching, the mothers of Case 1 and Case 2 showed scores in the typical range at T0 and above the typical range at T1 and T2. A significant improvement was observed in the mother of Case 4, who initially scored zero at T0 and subsequently achieved scores within the typical range at T1 and T2. A slight improvement was also evident in the mother of Case 3, which showed scores below the typical range at T0 and T1 and a score in the typical range at T2, although scores remained lower than those of the other dyads.

**Table 3:** Scores of PICCOLO domains for mothers of the four infants at the three time points (⇑ indicates an improvement of ≥5 points compared to T0; ⇓ indicates a decrease of ≥5 points compared to T0; = indicates a change of <5 points compared to T0).

	Dyad 1			Dyad 2			Dyad 3			Dyad 4		
PICCOLO	T0	T1	T2	T0	T1	T2	T0	T1	T2	T0	T1	T2
**Affection**	13	7	12	13	11	11	4	1	4	14	14	14
**Responsiveness**	12	10	12	9	11	12	5	1	4	4	6	11
**Encouragement**	10	9	12	5	9	12	4	1	4	3	5	9
**Teaching**	7	12	11	8	9	12	2	2	4	0	9	8
**Total Score**	42	38 =	47 ⇑	35	40 ⇑	47 ⇑	15	5 ⇓	16 =	21	34 ⇑	42 ⇑

### 3.5. Scores at Satisfaction Questionnaire

The mean score across the 15 items of the questionnaire was calculated for Cases 2, 3, and 4. Parents of Cases 2 and 3 reported very high satisfaction with the intervention provided between T0 and T1 (Case 2: 4.7/5; Case 3: 4.2/5). Parents of Case 4 reported a mean score of 3.7/5, indicating good satisfaction.

## 4. Discussion

The present study aimed to examine the developmental trajectories of three infants at HR for neurodevelopmental disorders and one LR infant for one year during the implementation of a very early multidimensional intervention. Specifically, we analyzed the outcomes of these infants, including their motor, cognitive, communicative, and socio-emotional development, as well as parental interaction styles. The most valuable result is the notable differences in developmental trajectories between the four infants, with significant variation in the effectiveness of interventions. In the LR child (Case 1), no signs of concern were detected in developmental assessments, and the child showed stable maturation of skills across all outcome measures. This infant was considered at LR because he is a sibling of a typically developing child. Consistent with this expectation, this outcome underlines the utility of the Griffiths-3 and CSBS-IT-C as reliable measures for tracking typical development before the first year of age. The analysis of parent–child interactions confirms the adequate functioning of the dyad since the mother maintained good levels of affection, responsiveness, encouragement, and teaching, which may have contributed to the stable developmental trajectory of this child.

In contrast, the HR children showed a mixed response to early interventions, although good-to-high satisfaction levels from parents are reported in all three cases. Case 2 was considered as HR because of being a sibling of an ASD child. His behavior was classified as “no concern” at baseline evaluations, as there were no early signs of neurodevelopmental vulnerability on the Griffiths-3 and CSBS-IT-C measures. For this reason, he was enrolled in the AM group, where the child and their parents are involved in parent-coaching sessions once/a month for six months. This child demonstrated steady development in the various outcome measures, with no significant score decline. This suggests the potential benefit of early active monitoring for HR children, even when no immediate developmental concerns are observed. Although the intervention does not appear to have had an impact on the child’s trajectory, it may have provided a protective environmental factor against later developmental delays, as the literature suggests that HR children, despite no early signs of concern, may be at an increased risk for developing other neurodevelopmental disorders [18]. This is also supported by the gradual improvement in responsiveness, encouragement, and teaching behaviors of the mother of Case 2, which suggests that the Active Monitoring intervention had a sound effect on the parents’ approach to their child’s development. Moreover, although data from all outcome measures were in the typical range, it is evident that this child shows different functioning, especially in social communicative skills, compared with the LR infant (Case 1), as shown particularly by CSBS-IT-C results. This may confirm the fact that in the absence of a clear diagnosis, siblings of ASD children show differences in social functioning, compatible with a biological basis of neurodivergence in these families [11]. Based on these issues, these findings support the importance of interventions targeting both the child and the parent, which can yield beneficial outcomes for children’s trajectories.

The findings for Case 3 were less encouraging. Despite receiving a weekly early intervention for six months, since some signs of concern were evident from baseline evaluations, the child’s developmental trajectory did not show any improvement. Instead, he exhibited a progressive deflection of the trajectories of communicative and cognitive skills, and all developmental domains fell within the low levels for the chronological age at all time points. This finding may suggest that this child could be part of the group of children with persistent high/moderate severity of symptoms, as described for ASD subjects [30,31]. Moreover, these data support the idea that the child’s general level of ability pre-intervention (in particular for language, motor, and intellectual functioning) could be a significant moderator of the effect of the intervention, as already suggested by other studies [32,33]. However, data about this child must be analyzed, considering jointly the parents’ behavior. As a matter of fact, data from PICCOLO showed consistently low levels of engagement of the mother in all areas. This is in line with the results of Apicella et al. [34], on retrospective analysis of family movies, who found that, although the time that ASD and TD dyads spent in involvement and responsiveness did not differ, opposite trajectories of interaction modalities and reciprocity already emerged during the first year of life. Indeed, the dyads with infants later diagnosed with ASD showed behaviors characterized by the absence of growth in the infants’ responsiveness and by the parallel decrease in the time their caregivers spent attracting their attention to engage them in an interaction. Progressively, caregivers of infants with ASD, compared to caregivers of TD infants, showed a significant reduction in affective touch. In contrast, the time spent on stimulating gestures increased, suggesting that this asynchrony between the caregiver and their infant may be under construction since the first year of life [34]. Disruption to this delicate equilibrium, resulting from developmental challenges in the child, has the potential to contribute to these discrepancies. Although the subject of our study falls within a complex NDD trajectory, we hypothesize that the previously explained mechanism can be applied to this dyad.

Finally, early intervention provided to Case 4, another HR child with developmental concerns at baseline, resulted in suggestive improvements across all developmental domains. Notably, Case 4′s scores on the Griffiths-3 moved from below the typical range to within normal limits, particularly in language, communication, and eye–hand coordination skills. These findings are consistent with prior research suggesting that early, targeted interventions tailored to the child’s profile can improve developmental outcomes. Moreover, in Case 4, significant changes in parenting behaviors were observed, with an increasing score, particularly in the domains of encouragement and teaching. These attitudes may have contributed to the child’s developmental progress, as demonstrated by the correlation between positive parental engagement and better developmental outcomes in children [17]. Furthermore, the potential for differences in the baseline levels of affective behaviors exhibited by mothers of Cases 3 and 4 to have a role in this phenomenon cannot be discounted. Higher scores in this domain of parenting behavior could have positively impacted the efficacy of parent coaching, thereby strengthening compliance and sustaining the child’s development. However, it should be noted that this assertion is based on individual cases and will require a larger sample to be rendered meaningful.

So, on the one hand, the importance of including parents in the intervention process, especially in HR children, is evident because parental behaviors can be key mediators of developmental progress. On the other hand, it is unlikely that differences in symptom severity change are determined exclusively by differences in intervention history, particularly in children who show global stability of the severity of symptoms, as already reported in the literature [31].

## 5. Limitations

This study has several limitations that should be acknowledged. First, its design—a report of four clinical cases—limits the generalizability of the findings. However, the cases were selected to exemplify potential developmental trajectories observed in siblings of children with ASD in clinical settings. Second, only male participants were included. This choice was made to maximize comparability across cases and because ASD is more frequently diagnosed in males, as consistently reported in the literature. Finally, further analyses on larger samples, including comparisons with control groups and long-term follow-up data, are warranted. Despite these limitations, the present findings may offer valuable insights for clinicians and researchers involved in early intervention programs for children at risk of neurodevelopmental disorders.

## 6. Conclusions

Early intervention for children at high risk of neurodevelopmental disorders can significantly improve developmental outcomes and lessen long-term impacts. Despite advances in detection and intervention, challenges such as accessibility and family engagement remain, particularly when atypical developmental signs are not evident in the first year. For these reasons, policies are urgently needed to enhance access to family-centered programs for at-risk infants. In this context, it is also important to underline the need to implement very early interventions that consider the child according to a global approach, enriching all developmental domains and not only specific sections of skills. This aspect has to be also considered in the view that, in typically developing children, early gross-motor, prelinguistic, and social developments show trackable idiosyncratic trajectories and also that typically maturity in gross motor performance links to concurrent prelinguistic and social development [35].

While this is a case series report, this study examines developmental measures and parental interactions, considering genetic and environmental influences on outcomes. Broader assessments of developmental outcomes may provide insights into the long-term effects of early interventions. In particular, the ongoing ERI-SIBS project [13] has the potential to study, using a controlled trial approach, the feasibility and the effect of this type of intervention for early infants at risk of neurodevelopmental disorders, providing behavioral, technological, and biomolecular data. The translational framework of the ERI-SIBS study is proving both feasible and highly promising. Its success relies on national health service clinicians specifically trained in early childhood development and neurodevelopmental disorders. The extensive data being collected are expected to yield critical insights into the social impact of early intervention on families of children with ASD, thereby advancing our understanding of the mechanisms, timing, and predictive markers underlying its effectiveness. Future research should increasingly focus on identifying health determinants affecting developmental trajectories and developing individualized intervention models. Emphasizing preventive care and community-based support for families is crucial, and further studies with larger samples and longer follow-ups are needed to refine strategies for high-risk infants.

## Figures and Tables

**Table 1:** Main clinical data of the four recruited children.

	Case 1	Case 2	Case 3	Case 4
Risk	Low	High	High	High
ERI-SIBS Group	Group 1 Clinical Monitoring	Group 2 Active Monitoring	Group 3 Early Intervention	Group 3 Early Intervention
Gestational Age (weeks)	40	40	39	38
Birth Weight (g)	3600	2950	3150	3105
Sex	Male	Male	Male	Male
Age of parents (Mother/Father)	39 ys/43 ys	40 ys/43 ys	28 ys/39 ys	35 ys/42 ys
Age at recruitment (months)	8	7	7	7
Age at the start of intervention (months)	---	9	9	8, 5
Griffiths-III GDQ at T0	113	114	79	82

**Table 2:** Subscales and Global Developmental Quotients on the Griffiths-3 of the four children during the year of participation in the project (⇑ indicates an improvement of >5 points compared to T0; ⇓ indicates a decrease of >5 points compared to T0; = indicates a change of ≤5 points compared to T0).

	Case 1			Case 2			Case 3			Case 4		
Griffiths-III	T0	T1	T2	T0	T1	T2	T0	T1	T2	T0	T1	T2
**Foundation of Learning**	109	105 =	119 ⇑	128	100 ⇓	110 ⇓	91	76 ⇓	48 ⇓	93	105 ⇑	92 =
**Language and Communication**	125	115 ⇓	104 ⇓	117	109 ⇓	104 ⇓	78	81 =	69 ⇓	85	106 ⇑	96 ⇑
**Eye–Hand Coordination**	98	120 ⇑	113 ⇑	106	126 ⇑	104 =	92	71 ⇓	52 ⇓	95	109 ⇑	105 ⇑
**Personal–Social–Emotional**	105	111 ⇑	107 =	119	123 =	114 =	76	54 ⇓	75 =	78	110 ⇑	96 ⇑
**Grossmotor**	122	114 ⇓	113 ⇓	107	128 ⇑	118 ⇑	86	67 ⇓	74 ⇓	75	119 ⇑	74 =
**Global Developmental Quotient**	119	116 =	113 ⇓	114	116 =	113 =	79	61 ⇓	57 ⇓	82	106⇑	90 ⇑

## Data Availability

Due to privacy and ethical restriction the data presented in this study are available on request from the corresponding author.

## Abbreviations

ASD	Autism Spectrum Disorder
NDD	Neurodevelopmental Disorder
ERI-SIBS	Early Recognition and Intervention in SIBlingS at High Risk for Neurodevelopment Disorders
CM	Clinical Monitoring
AM	Active Monitoring Group
EI	Early Intervention
CSBS-IT-C	Communication and Symbolic Behavior Scales—Infant–Toddler Checklist
ADOS-2	Autism Diagnostic Observation Schedule, Second Edition
PICCOLO	Parent Interactions with Children: Checklist of Observations Linked to Outcomes
TNPEE	Neuro and psychomotor therapist for developmental age
